# Beyond “Fire” and “Ashes”: The Influence of Trait Characteristics on the Response to Mood Stabilizers in Bipolar Disorders

**DOI:** 10.3390/brainsci15050490

**Published:** 2025-05-07

**Authors:** Alfonso Tortorella, Francesca Scopetta, Gianmarco Cinesi, Ilaria Baldini, Antonio Russo, Kety Amantini, Filippo De Giorgi, Giulia Menculini

**Affiliations:** 1 Section of Psychiatry, Department of Medicine and Surgery, University of Perugia, 06132 Perugia, Italy; francesca.scopetta@yahoo.it (F.S.); gianmarco.cinesi@gmail.com (G.C.); baldini.ilaria95@gmail.com (I.B.); giulia.menculini@unipg.it (G.M.); 2 Psychiatric Inpatient Unit (S.P.D.C.), Department of Mental Health, Local Health Unit USL Umbria 1, 06129 Perugia, Italy; ant.russo88@gmail.com (A.R.); kety.amantini@uslumbria1.it (K.A.); 3 Division of Psychiatry, Department of Neuroscience and Sensory Organs, General Hospital of Perugia, 06129 Perugia, Italy; filippo.degiorgi@ospedale.perugia.it

**Keywords:** bipolar disorder, mood stabilizers, treatment response, impulsivity, affective temperaments

## Abstract

*Background:* The present study aimed to investigate the clinical correlates of treatment response to mood stabilizers in patients with bipolar disorder (BD), with a specific focus on trait-related characteristics such as impulsivity and affective temperaments. *Methods:* In- and outpatients diagnosed with BD were recruited at the Section of Psychiatry of the General Hospital/University of Perugia. Socio-demographic, clinical, and current psychopathological characteristics were collected. The treatment response was retrospectively assessed using the Alda Scale. Trait characteristics were evaluated through the Barratt Impulsiveness Scale (BIS-11) and the Brief Temperament Evaluation of Memphis, Pisa, and San Diego—Münster version (briefTEMPS-M). Bivariate analyses and a general linear model were employed to analyze the correlates of treatment response to mood stabilizers. *Results:* Among the investigated variables, trait impulsivity showed a significant negative association with treatment response. A similar effect was observed for depressive temperament, while other affective temperaments were not significantly associated with treatment outcomes. Patients with good treatment responses exhibited higher illness duration and lower severity of BD, higher prevalence of comorbid anxiety disorders, lower diurnal variation in depressive symptoms, and lower functional impairment in autonomy and occupational domains. The main limitations of this study were represented by the small sample size, the retrospective assessment of treatment response, and the inclusion of patients from a single center. *Conclusions:* The present findings suggest that impulsivity and depressive temperament should be investigated as potential predictors of poor response to mood stabilizers in BD. These trait dimensions, together with other clinical markers, may serve as useful targets for patient stratification and the development of personalized treatment strategies.

## 1. Introduction

Mood stabilizers are a cornerstone in the treatment of bipolar disorder (BD), playing a crucial role in shaping its clinical course. Despite their established efficacy, a considerable proportion of patients fail to achieve an adequate clinical response, resulting in suboptimal outcomes and persistent functional impairment [1].

Lithium is the mood stabilizer with the most robust clinical evidence and is considered a first-line therapeutic strategy for BD acute treatment and prophylaxis, mainly due to its efficacy in preventing depressive and manic episodes and maintaining mood stability. Nevertheless, only approximately 30% of patients with BD demonstrate an optimal response to lithium monotherapy, highlighting substantial heterogeneity in treatment outcomes [2], possibly influenced by a combination of clinical, psychosocial, and genetic factors [2,3].

Previous evidence indicates that a favorable lithium response is more common in patients with type I BD (BDI) compared to type II BD (BDII) and that lithium responders predominantly belong to a clinical subtype characterized by euphoric manic episodes and melancholic depressions, manic–depressive interval (MDI) cycling patterns, absence of rapid cycling, lower prevalence of psychiatric comorbidities and psychotic symptoms, and a more frequent familial history of mood disorders [4,5,6,7].

Research on broader classes of mood stabilizers remains limited. Recent advances in precision psychiatry have emphasized the need to tailor treatment strategies in BD based on individual clinical features, which can guide personalized therapeutic approaches [8,9,10]. The strong need for targeted treatment strategies has been supported by the application of machine learning algorithms to real-world data, which may enhance our ability to predict treatment response in BD, marking a significant step toward precision psychiatry [11]. Different clinical characteristics were listed as possible contributors. In particular, previous studies identified a family history of substance abuse, childhood trauma, higher frequency of mood episodes, and comorbid substance abuse as predictors of poor treatment outcomes [12,13,14]. Circadian rhythm disruptions and chronotypes further influence treatment response in BD since responders more often exhibit morning chronotypes, while an evening chronotype is associated with greater clinical severity and poorer response, potentially due to underlying biological circadian misalignments [15,16,17].

Moreover, stable traits including impulsivity and affective temperaments may significantly contribute to individual variability in treatment responses and clinical outcomes in BD. Trait impulsivity is consistently elevated in BD and is associated with higher illness severity, increased hospitalization rates, reduced medication adherence, and higher suicide risk, negatively influencing long-term outcomes [18,19]. Among trait characteristics, affective temperaments—stable subclinical manifestations or phenotypes influencing mood disorder presentations—are also critical in BD prognosis [20]. Cyclothymic and irritable temperaments are associated with poorer clinical outcomes, higher relapse rates, increased aggression, and suicidality, whereas hyperthymic temperament has been found to confer protective effects, predicting fewer relapses and milder symptomatology [21]. A depressive temperament similarly predicts poorer medication adherence and overall treatment outcomes, suggesting the need for tailored interventions addressing adherence and self-management skills [19]. Since trait characteristics can be detected early during an illness course, the identification of specific features associated with treatment response would be of great importance for the implementation of further strategies aimed at the optimization of outcomes in BD.

Given this background, the present study aimed to identify clinical correlates associated with mood stabilizer treatment response in patients with BD, focusing on trait-related characteristics such as impulsivity and affective temperaments. We hypothesize that these variables exert significant influence on treatment response to mood stabilizers and that the characterization of this association may be useful in the clinical practice to enhance patient stratification and personalize treatment strategies.

## 2. Materials and Methods

The present cross-sectional study was conducted at the Section of Psychiatry of the General Hospital/University of Perugia. Both in- and outpatients aged ≥18 years, diagnosed with BD according to the latest version of the Diagnostic and Statistical Manual of Mental Disorders (DSM) [22,23], and willing to participate in this study were recruited. Subjects were excluded if unable to provide written informed consent, in case of at least moderate cognitive impairment, comorbidity with any medical condition potentially affecting their psychopathological state, and/or insufficient comprehension of the Italian language (oral/written). All subjects meeting the inclusion criteria received a comprehensive explanation of the study aims and design and provided their written informed consent for study participation. Psychiatric diagnoses were assessed using the Structured Clinical Interview for DSM-5 Disorders, Clinician Version (SCID-5-CV) [24]. This study was conducted according to the latest version of the Declaration of Helsinki and received approval from the Ethics Committee of the Umbria Region (12,958/18/ON, update N. 21,256/21/ESS).

Socio-demographic and clinical characteristics, including main diagnosis, familiar psychiatric history, psychiatric and medical comorbidities (including substance/alcohol use), age at onset, duration of untreated illness, number of episodes, predominant polarity, number of hospitalizations, seasonality, lifetime suicide attempts, mixed features according to DSM-5 specifier, psychotic symptoms, aggression, and response to antidepressant treatment, were collected using an ad hoc schedule by clinical interviews with the included patients. Age at onset was retrospectively assessed as the age of the first affective episode, either depressive or hypomanic/manic. Duration of untreated illness was evaluated by subtracting the age at onset from the age at the first appropriate treatment for BD. Predominant polarity was defined when at least two-thirds of affective episodes belonged to the same polarity [25].

The included patients underwent a psychiatric assessment for the evaluation of the overall severity of BD (Clinical Global Impressions—Bipolar Disorder Version [26]), of depressive (Hamilton Rating Scale for Depression—HRSD [27]), manic (Mania Rating Scale—MRS [28]), and psychotic (Positive and Negative Syndrome Scale—PANSS; [29]) symptoms, of the current severity of circadian rhythm disruptions (Biological Rhythms Interview for Assessment in Neuropsychiatry—BRIAN [30]), and of chronotype (Morningness–Eveningness Questionnaire—MEQ [31]). Global functioning was evaluated with the Functioning Assessment Short Test (FAST; [32]).

As for trait characteristics, affective temperaments were evaluated using the self-administered Brief Temperament Evaluation of Memphis, Pisa, and San Diego—Münster version (briefTEMPS-M) scale [33,34], comprising 35 items assessing 5 affective temperaments: depressive, cyclothymic, hyperthymic, irritable, and anxious. The briefTEMPS-M does not have defined cut-off scores, so the mean scores were analyzed for each temperament [34]. We used the Barratt Impulsiveness Scale, 11 items, (BIS-11) [35,36] to evaluate impulsiveness as a trait characteristic. The BIS-11 is an auto-administered questionnaire composed of 30 items, assessing different dimensions of impulsivity, particularly attentional, motor, and non-planning impulsivity. The two scales were always completed by the included patients in the presence of psychiatrists, with expertise in mood disorder evaluation who were trained for the study procedure, to ensure proper comprehension and compliance.

The response to mood stabilizer treatment was retrospectively assessed using the Alda Scale, a clinician-administered instrument that considers two criteria. Criterion A evaluates clinical improvement with mood stabilizer treatment, while criterion B rates the degree of causal relationship between clinical improvement and mood stabilizer treatment. The total score is calculated by subtracting score B from score A. The scores range from a 0 to 10 scale, where a score ≥ 7 indicates a good response, a score from 4 to 6 a moderate response, and a score of ≤3 a poor response, as recommended by previous studies, suggesting the use of the scale for stratifying BD samples according to treatment response [37,38]. The Alda Scale is considered to be a valid measure, with interrater reliability of 0.54–0.75 in assessing long-term response to treatments [37,38].

The collected information was entered into an electronic dataset created with the Statistical Package for Social Sciences (SPSS), version 26. There was no missing data concerning the variables of interest. Descriptive analyses were performed to assess the distributional properties of the variables of interest in the sample; categorical variables were expressed as absolute frequencies and percentages, while continuous variables were described by using the mean as the measure of centrality and the standard deviation as the measure of dispersion. Continuous variables were considered as normally distributed according to the central limit theory [39]. Patients were then divided into two subgroups according to the evidence of a good treatment response to mood stabilizers according to the Alda Scale. Between-group comparisons were performed using the Chi-square or the Student’s *t*-test depending on the type of variable. All tests were two-tailed, with a level of significance set as *p* < 0.05. A univariate General Linear Model (GLM, UNIANOVA) was employed to explore the impact of the considered trait characteristics on treatment response. The dependent continuous variable was the Alda total score. Fixed factors included biological sex and the presence of comorbid personality disorders. Covariates comprised age, the BIS-11 total score, and the five briefTEMPS-M subscores. Biological sex, age, and the presence of comorbid personality disorders were included as possible confounding factors influencing both treatment response and other trait characteristics. The status of the patient (in/outpatient) at the moment of evaluation was included in the model as a random factor to account for variability between in- and outpatient groups. Prior to conducting GLM analyses, we evaluated the assumptions of normality and homoscedasticity. The normality of residuals was assessed through visual inspection of Q-Q plots and confirmed via the Shapiro–Wilk test. Homoscedasticity was examined using Levene’s test for equality of variances. These diagnostics indicated that the assumptions were sufficiently met for the main outcome variables. A significance level was set at 0.05, and partial eta squared (η^2^) was calculated to estimate effect sizes, with values greater than 0.06 interpreted as moderate effects. We calculated and reported 95% confidence intervals for each η^2^_p_ estimate to enhance the interpretability and robustness of our findings.

## 3. Results

Our sample consisted of 91 patients diagnosed with BD, most of whom were females (n = 57, 62.6%), with a mean age of 43.82 ± 15.32 years. The predominant diagnosis was BDII (n = 48, 52.7%). Most subjects were inpatients (n = 77, 84.6%) and were evaluated during a manic or hypomanic episode (n = 41, 45.1%). The socio-demographic characteristics of patients are presented in Table 1.

In our population, most patients were treated with lithium (n = 33, 36.3%) or valproic acid (n = 32, 35.2%) monotherapy. The mean Alda Scale score was 3.7 ± 3.2 (range 0–10; median 4, IQR 7), with 27 patients (29.7%) classified as good treatment responders. Subjects with a good treatment response did not differ significantly from those who did not respond adequately to mood stabilizer treatment regarding socio-demographic characteristics, psychiatric family history, and medical comorbidities. As for clinical course features, patients with a good response had a longer disease duration than those with a poor response (14.11 ± 2.77 vs. 12.81 ± 1.65 years, *p* = 0.034) and showed a higher prevalence of comorbid anxiety disorders (32% vs. 13.3%, *p* = 0.048) (see Table 2). Concerning psychopathological characteristics, patients with a poorer treatment response exhibited greater diurnal variation in depressive symptoms as measured by the HAM-D diurnal variation factor (1.09 ± 1.48 vs. 0.58 ± 1.08, *p* = 0.045) and greater severity of BD as assessed by the CGI-BP scale (4.26 ± 1.57 vs. 3.77 ± 0.87, *p* = 0.049). Analysis of overall functioning using the FAST scale showed that patients with a good treatment response had less impairment in autonomy (3.32 ± 3.37 vs. 5.48 ± 3.95, *p* = 0.025) and occupational functioning (5.27 ± 4.83 vs. 7.63 ± 4.87, *p* = 0.047).

The GLM analysis revealed statistically significant effects of impulsivity (F = 5.18, *p* = 0.027; η^2^ partial = 0.097, 95% CI = −0.200, −0.012) and depressive affective temperament (F = 4.70, *p* = 0.035; η^2^ partial = 0.089, 95% CI = −0.423, −0.016) on the Alda total score, suggesting that these trait variables significantly influence mood stabilizer treatment response with moderate effect sizes. Standardized regression coefficients (BIS-11 total score: Beta = −0.153; briefTEMPS-M depression: Beta = −0.260) pointed out a negative association between the BIS-11 and briefTEMPS-M scores and the Alda Scale total score. No statistically significant effects were found for cyclothymic, hyperthymic, irritable, or anxious temperaments, age, biological sex, presence of personality disorders, or treatment status (all *p* > 0.05).

## 4. Discussion

The present study investigated the clinical correlates of the response to mood stabilizers in patients with BD. Our findings highlight the significant contribution of impulsivity and affective temperaments, particularly depressive temperament, to treatment outcomes, suggesting that these stable trait dimensions present a negative association with treatment response and might thus hold prognostic relevance in clinical practice. These results align with the previous literature indicating that higher impulsivity correlates with treatment resistance, reduced medication adherence, higher drop-out rates, and more frequent relapses in BD [40]. Specifically, non-planning impulsivity was significantly associated with poor medication adherence even in euthymic patients, possibly fostering disorganized behaviors and inconsistent treatment engagement and negatively impacting therapeutic response and overall clinical outcomes. Thus, impulsivity may represent a potential early therapeutic target or stratification factor for personalized interventions. Notably, impulsivity is a hallmark in the clinical presentation of BD, leading to a worse disease course and higher suicide rates [41]. Furthermore, altered risk-taking behaviors have been observed in patients with early-stage BD and a history of psychosis, emphasizing the complexity of impulsivity-related manifestations in different subgroups [42]. Consequently, tailored strategies combining pharmacological and psychosocial treatments should be proposed for patients displaying high impulsivity rates even since illness onset.

Depressive temperament also negatively influenced treatment response, emphasizing the importance of affective temperaments in BD characterization and management. Depressive temperament is typically associated with chronic subthreshold depressive symptomatology, lower response rates to standard pharmacological treatments, and greater functional impairment. Clinical studies have found that patients with depressive predominant polarity, which has been linked to longer affective episode duration, more frequent suicide attempts, and conducts of abuse [43], often present with an underlying depressive temperament [44]. Patients with depressive or cyclothymic affective temperaments also showed significantly poorer functional outcomes following a manic episode compared to those with hyperthymic temperaments [45]. Baldessarini et al. [46] also reported that depressive temperament predicts poorer treatment response in mood disorders, further supporting the hypothesis that a depressive temperamental background predisposes individuals to longer, less treatment-responsive depressive episodes. Depressive symptoms represent the most challenging clinical features for BD treatment, significantly influencing quality of life and functioning [47]. These manifestations may necessitate combined treatments with antidepressants, increasing overall complexity due to difficulties in treatment adherence and phenomena such as treatment-emergent affective switches and worsening of depression [48,49], highlighting the need for personalized treatment strategies. Additionally, emotion dysregulation, a characteristic of atypical depression, further complicates clinical management and therapeutic responses, indicating potential additional targets for intervention [50].

Significant differences were found when performing between-group comparisons. Good responders exhibited longer illness duration and a higher prevalence of comorbid anxiety disorders. As for illness duration, this feature was poorly investigated since most studies focused on DUI in BD [14,51]. Our findings may reflect a cumulative stabilization effect over time, due to different treatment attempts, or indicate that patients with a more episodic and defined course benefit more from mood stabilizers. The higher prevalence of comorbid anxiety disorders in responders is an intriguing finding, seemingly contrasting with some of the previous literature, which generally associates anxiety with a worse prognosis [52]. Anxiety disorders commonly affect 30–50% of patients with BD, significantly impacting medication adherence and clinical outcomes [53]. This comorbidity frequently necessitates complex pharmacological regimens and may exacerbate perceived side effects, possibly leading to reduced adherence. Anyway, previous findings already underlined higher treatment gains in BD patients with comorbid anxiety, with a greater response to non-pharmacological interventions [54]. Moreover, some mood stabilizers, e.g., sodium valproate and lamotrigine, were demonstrated to be effective for the treatment of anxiety symptoms in BD [55]. Another possible explanation of our findings is that BD patients with comorbid anxiety may experience higher levels of subjective distress, leading them to seek help more promptly and adhere more strictly to prescribed treatments. This heightened symptom awareness and proactive treatment-seeking behavior could facilitate earlier intervention and more consistent pharmacological adherence, ultimately improving outcomes [56]. To note, some stable characteristics, particularly trait anxiety, may be associated with an increased risk of developing anxiety disorders [57]. Elevated trait anxiety not only predisposes individuals to the development of anxiety disorders but may also amplify their severity and persistence once established. In this perspective, despite anxious temperament being not associated with treatment response in this study, a further investigation of trait anxiety may help provide further insight into this topic in the future.

Patients with poorer treatment response exhibited significantly greater diurnal variation in depressive symptoms and increased overall illness severity as measured by CGI-BP, aligning with evidence emphasizing circadian disturbances as markers of treatment resistance. Circadian rhythm abnormalities, including irregular sleep–wake cycles and dysfunctional chronobiology, are frequently observed in BD and associated with treatment-resistant illness forms. All bipolar patients exhibit some degree of circadian disruption during acute episodes, and this biological instability may hinder remission [58]. Circadian dysregulation may increase vulnerability to mixed states and treatment-resistant depression. Some authors propose biological rhythm misalignment may reduce antidepressant efficacy and contribute to non-response in bipolar depression, supporting the role of biological clocks in therapeutic resistance [59]. Interventions targeting circadian mechanisms, such as light therapy and social rhythm therapy, have shown benefits, underscoring circadian disturbances as potential therapeutic targets.

Good responders demonstrated notably better functional outcomes, showing less impairment in autonomy and occupational functioning assessed via the FAST scale. When treatment achieves stable remission, significant improvements occur in psychosocial domains such as employment, relationships, and daily activities. Conversely, untreated BD is marked by low inter-episode functional recovery [60]. Achieving euthymia pharmacologically improves global functioning, although residual deficits may persist. Poor treatment response, whether due to non-adherence or pharmacological resistance, is associated with negative functional outcomes, including rehospitalization, unemployment, relational difficulties, and reduced autonomy [61]. Functional impairment may persist even after symptomatic remission in some cases, suggesting clinical response alone might not fully restore social and occupational functioning. Thus, optimizing both pharmacological and psychosocial interventions is essential for improving broader functional reintegration.

Beyond the associations observed between affective temperaments, impulsivity traits, and treatment response in BD, it is important to consider that these relationships may rely on further potential mediators or moderators. Treatment adherence has been demonstrated to critically affect clinical outcomes in BD and affective temperaments, e.g., cyclothymic or irritable temperaments, may be associated with lower adherence due to fluctuations in motivation, insight, or behavioral instability [62]. Similarly, impulsivity may influence compliance with pharmacological regimens, particularly when mood stabilizers require sustained and regular intake [63]. Furthermore, psychosocial factors, including the availability of social support, the presence of psychosocial stressors, and individual coping mechanisms, may act as moderators. Indeed, these factors may predispose to maladaptive interpersonal dynamics or chronic stress exposure, influencing the response to pharmacological treatment due to different mechanisms, also buffering against adverse events [64]. Considering these variables helps frame our findings within a broader biopsychosocial model and highlights the importance of a multidimensional assessment in both research and clinical practice.

The present study has limitations. First, the small sample size may limit the generalizability of findings, also preventing further between-group comparison and analysis stratification. Second is the use of a retrospective measure for the assessment of treatment outcomes. Indeed, despite being well validated and largely used in previous studies on subjects with BD, the retrospective investigation of treatment response constrains our ability to establish causal relationships between assessed traits and treatment outcomes due to potential recall biases. Subsequently, we acknowledge that our findings, while indicative, should be validated in prospective longitudinal studies designed explicitly to overcome these biases. We are also aware that the inclusion of one single center based in a university hospital rather than in primary care settings may not be fully representative of the population of people suffering from BD. Finally, it should be underlined that several variables may influence treatment response in mood disorders. In particular, treatment adherence and socio-economic status were not systematically assessed in our study—except for one specific question in the Alda Scale considering adherence to medications during the period of clinical stability—and should hopefully be included in future research.

## 5. Conclusions

The present study highlights the clinical relevance of trait impulsivity and depressive temperament in predicting treatment responses to mood stabilizers in BD. Our findings suggest that higher impulsivity and predominant depressive temperament negatively influence therapeutic outcomes, contributing to poorer medication adherence, increased illness severity, and functional impairment. Further clinical features, such as circadian rhythm disturbances, may increase overall complexity, underscoring the need for personalized therapeutic strategies. Future research should investigate trait characteristics as potential targets for early intervention, ultimately aiming to optimize individualized care and improve long-term prognosis in patients with BD.

## Figures and Tables

**Table 1:** Socio-demographic characteristics of patients in the sample.

Socio-Demographic Variable	Absolute Frequency	Percentage
Woman	57	62.6
Italian Nationality	85	93.4
Education Level > High School	19	20.9
Currently Married	30	33.3
Single	58	63.7
Ever Worked	58	63.8
Currently Employed	40	44
Living Alone or with Marital Family	59	64.8
	**Mean**	**Standard Deviation**
Age	43.82	15.32

**Table 2:** Comparison of clinical and course characteristics between patients who responded to treatment (n = 27, 29.7%) and those who did not (n = 64, 70.3%).

	Responders (%)	Non-Responders (%)	χ^2^	*p*
BDII	47.8	30.8	1.341	0.247
Familiar psychiatric history	69.2	50.8	1.819	0.177
Medical comorbidities	57.7	50	0.183	0.668
Long DUI	50	52.5	0.000	1.000
Depressive onset	55.6	51.6	0.012	0.911
Predominant polarity	86.4	77.6	0.317	0.573
Psychotic symptoms	46.2	40.7	0.054	0.816
Depressive atypical features	16.7	21.1	0.022	0.883
Mixed features	30.8	29.5	0.000	1.000
Aggressive behaviors	32	31.6	0.000	1.000
Seasonality	29.6	27.9	0.000	1.000
Suicide attempts	32	36.1	0.012	0.912
Comorbid anxiety disorders	32	13.3	2.895	**0.048**
Comorbid eating disorders	12	18	0.134	0.714
Comorbid ADHD	4	3.3	0.000	1.000
Comorbid personality disorders	26.9	38.6	0.618	0.432
Substance/alcohol abuse	25.9	40.6	1.196	0.274
Treatment-emergent manic switch	6.3	15.9	0.011	0.915
Evening chronotype	25	16.4	0.367	0.545
	**Responders (Mean, SD)**	**Non-Responders (Mean, SD)**	**t**	***p***
Age at onset	23.78 (10.25)	24 (10.15)	0.094	0.925
Illness duration	22.58 (14.11)	15.51 (12.81)	−2.279	**0.025**
Affective episodes (n)	10.29 (15.38)	7.87 (8.53)	−0.757	0.452

Legend: ADHD = Attention Deficit/Hyperactivity Disorder; BDII = bipolar disorder type II; DUI = duration of untreated illness; SD = standard deviation. Notes: Bold values indicate statistical significance (*p* < 0.05).

## Data Availability

The data presented in this study are available upon request from the corresponding author due to ethics and privacy reasons.
